# Influence of the Casting Concentration on the Mechanical and Optical Properties of FA/CaCl_2_-Derived Silk Fibroin Membranes

**DOI:** 10.3390/ijms21186704

**Published:** 2020-09-13

**Authors:** Alexander Kopp, Laura Schunck, Martin Gosau, Ralf Smeets, Simon Burg, Sandra Fuest, Nadja Kröger, Max Zinser, Sebastian Krohn, Mehdi Behbahani, Marius Köpf, Lisa Lauts, Rico Rutkowski

**Affiliations:** 1 Fibrothelium GmbH, 52068 Aachen, Germany; alexander.kopp@fibrothelium.com (A.K.); marius.koepf@fibrothelium.com (M.K.); lisa.lauts@fibrothelium.com (L.L.); 2 Department of Oral and Maxillofacial Surgery, University Medical Center Hamburg Eppendorf, 20251 Hamburg, Germany; schunck.laura@gmx.de (L.S.); m.gosau@uke.de (M.G.); r.smeets@uke.de (R.S.); s.burg@uke.de (S.B.); 3 Department of Oral and Maxillofacial Surgery, Division of Regenerative Orofacial Medicine, University Medical Center Hamburg-Eppendorf, 20251 Hamburg, Germany; s.fuest@uke.de; 4 Department of Plastic, Reconstructive and Aesthetic Surgery, University Hospital of Cologne, 52074 Cologne, Germany; nadja.kroeger@uk-koeln.de (N.K.); max.zinser@uk-koeln.de (M.Z.); 5 Polyclinic for Dental Prosthetics, University Medical Center Göttingen, 37075 Göttingen, Germany; sebastian.krohn@med.uni-goettingen.de; 6 University of Applied Sciences, FH Aachen, 52428 Jülich, Germany; behbahani@fh-aachen.de

**Keywords:** silk, membrane, fibroin, biopolymer, tissue regeneration

## Abstract

In this study, we describe the manufacturing and characterization of silk fibroin membranes derived from the silkworm Bombyx mori. To date, the dissolution process used in this study has only been researched to a limited extent, although it entails various potential advantages, such as reduced expenses and the absence of toxic chemicals in comparison to other conventional techniques. Therefore, the aim of this study was to determine the influence of different fibroin concentrations on the process output and resulting membrane properties. Casted membranes were thus characterized with regard to their mechanical, structural and optical assets via tensile testing, SEM, light microscopy and spectrophotometry. Cytotoxicity was evaluated using BrdU, XTT, and LDH assays, followed by live–dead staining. The formic acid (FA) dissolution method was proven to be suitable for the manufacturing of transparent and mechanically stable membranes. The fibroin concentration affects both thickness and transparency of the membranes. The membranes did not exhibit any signs of cytotoxicity. When compared to other current scientific and technical benchmarks, the manufactured membranes displayed promising potential for various biomedical applications. Further research is nevertheless necessary to improve reproducible manufacturing, including a more uniform thickness, less impurity and physiological pH within the membranes.

## 1. Introduction

The development of functional materials that also exhibit the capacity for interacting with biological systems poses one of the central challenges in biomedical engineering. Named materials may be derived from nature or may also be synthetically manufactured, such as synthetic polymers, metals or alloys [1]. More recently, proteins acquired from natural precursors, which themselves essentially prevail as structural and functional polymers, have been constituted as biomaterials. In this context, silk has gained significant attention among the scientific and clinical community due to its manyfold advantageous properties. As silk is naturally produced by certain insect and arachnid species, precedingly inheriting a long tradition within the textile industry, raw silk consists of two parallel fibroin fibers (hydrophobic and water insoluble core filaments), which are cohered by a top layer of sericin (amorphous, hydrophilic and water-soluble glue-like protein surrounding fibroin fibers) [2,3,4]. The fibroin fibers derived from the *Mulberry silkworm* (*Bombyx mori*; *B. mori*) hold tremendous tensile strengths of up to 740 MPa and are tougher than Kevlar, which itself is predominantly recognized as a benchmark material with regard to high-performance fiber technology [5,6]. Further key biological properties of fibroin include excellent biocompatibility, oxygen and water permeability, diminished immunogenicity and decelerated biodegradation. Various studies have already concluded a widespread acceptance of fibroin as a potential biomaterial by demonstrating that properly degummed and sterilized silk products inherit a degree of biocompatibility that may be compared to other established biomaterials, e.g., collagen [7,8,9]. The degradation properties of silk fibroin have recently been investigated in vitro and in vivo [10,11]. In biomedical engineering, silk is employed equally as a raw material in its unaltered condition, as well as in its regenerated state after dissolution and further processing into various forms and shapes. In addition to its being embraced in the textile industry, it may also be applied as a suture material or wound dressing, a drug carrier or as a further component in tissue engineering [12,13,14,15,16,17,18,19]. Fibroin regeneration and processing enables the production of different 2D and 3D matrices (e.g., sponges, hydrogels, films) that offer a wide range of applicability, particularly for the replacement of various human tissues, including vessels, nervous tissue, cartilage, bone, skin, and cornea [15,20,21,22,23,24,25]. However, only a small number of applications of fibroin have been investigated in clinical studies or have been approved and certified as a medical device so far.

A proper manufacturing process that enables suitable degumming (removal of the compound protein sericin), dissolution and regeneration of silk is a requirement for further biomedical applications in order to properly disintegrate silk fibroin into the desired structural components. Nowadays, different methods are available for degumming, such as enzymatic, soap-assisted or acid-assisted processing, among various others [26,27]. In the second step, the now purified fibroin is further dissolved by employing chaotropic agents (e.g., LiBr or CaCl_2_) and then dialyzed for several days before processing [28,29]. As opposed to raw silk, this reverse engineering approach enables biological and physical properties that may be significantly altered in various ways by degumming and regeneration [15,30,31]. Additionally, structural integrity and biodegradation characteristics may also be modified sustainably. Furthermore, the presented techniques offer high economical potential in terms of time and cost efficiency, which is especially relevant for the manufacturing of biomedical implants [28,29,32].

The aim of this study was to evaluate the production processes and the resulting properties of casted silk fibroin membranes fabricated via formic acid (FA) and calcium chloride (CaCl_2_) dissolution. In recent years there have been several works that have established this novel dissolving process using FA/CaCl_2_ as an integral part of the regeneration process of silk fibroin [33,34,35,36,37]. Though this technique has recently evolved and is now recognized as a fast and cheap manufacturing method, it has scarcely been investigated so far. The objective was to investigate whether the membranes fulfilled fundamental requirements necessary for their application in the field of biomedical engineering. The membranes were thus characterized with respect to their physical structure along with their mechanical and optical properties in dependence on the fibroin concentration used for casting. During the manufacturing process, which includes complex washing and neutralization processes, the silk fibroin solution exhibits different pH values. With respect to the intended biomedical application, specific pH levels may trigger tissue interaction and were thus represented as a crucial aspect of the study in addition to the above-mentioned material-related properties.

## 2. Results

### 2.1. Washing Process and pH Neutralization

**Figure 1:**
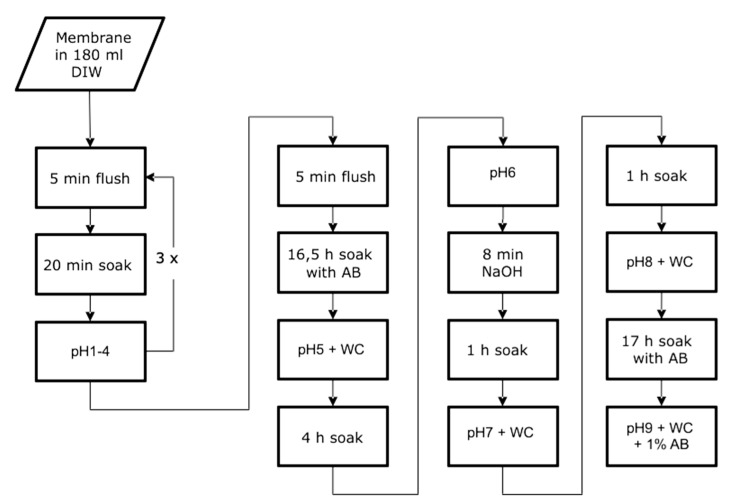
Flow chart depicting the washing and pH neutralization process for post-treatment during membrane production. DIW = Deionized water. AB = 1% Antibiotic (Pen Strep (Gibco Life Technologies, Carlsbad, CA, USA) with 10.00 units/mL Penicillin and 10.00 μg/mL Streptomycin). WC = Water change. Flush = Flush under running distilled water. Soak = Leave in 180 mL deionized water. pH = Measure pH value while stirring for 5 min. NaOH = Leave in 0.01 M NaOH solution.

pH values measured during washing and neutralization processes are displayed in Table 1. After neutralization, pH values increased significantly and reached values within the physiological range of 7.35 to 7.45. pH then again dropped significantly, as the membranes were exposed to antibiotic (AB) solution. The entire process lasted two days, and thus was an integral part of the manufacturing process (Figure 1). A significant relationship between the fibroin concentration of the starting solution and the pH value assessed during processing could not be detected.

### 2.2. Characterization of Surface and Structure

**Figure 2:**
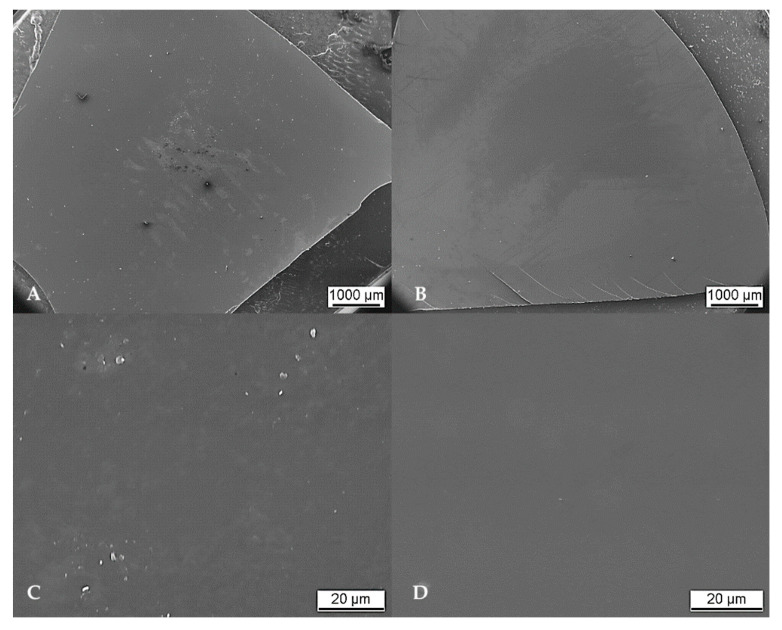
SEM images of SF10 membrane (**A**,**C**) and SF12 membrane (**B**,**D**) in low (**A**,**B**) and high magnification (**C**,**D**).

In addition to the photographs, the detailed surface structure was analyzed via scanning electron microscopy (SEM). Apart from negligible contamination by single particulates amounting to a few micrometers, the overall surface exhibited a smooth and homogenous appearance. Isolated cloudy areas revealed a different surface structure. These areas seemed as if “wiped off” and presumably represent residues generated throughout SEM preparation by gold sputtering. Isolated cracks found specifically at the edges of the membrane were thus related to cutting the membrane shape from the casted blanks. Significant differences in morphological appearance in relation to the respective fibroin concentration were not observed. An overall SEM overview and respective close-up images of SF10 and SF12 membranes are exemplarily displayed in Figure 2. SEM overview and cross-sectional images of all SF samples are added as Annex 1.

### 2.3. Determination of Membrane Thickness

**Figure 3:**
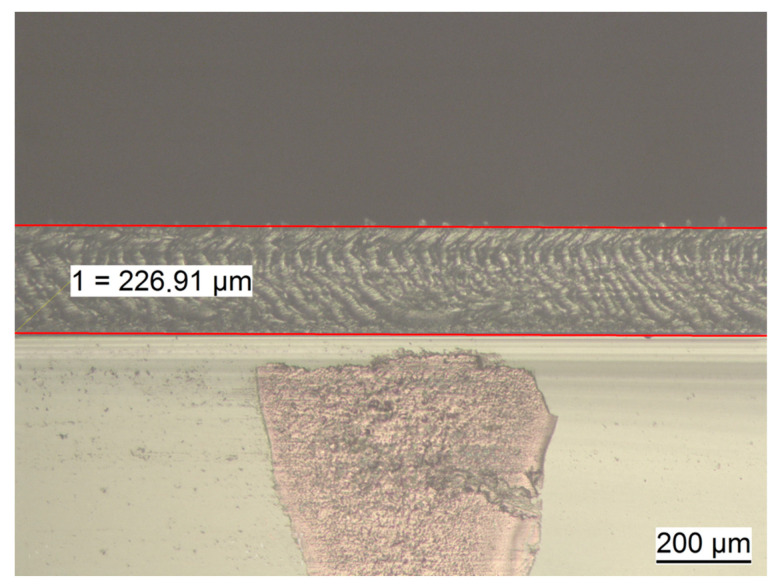
Thickness measurement procedure on a SF12 membrane. Thickness is determined by measuring the vertical distance between the two red parallel lines placed on the outer edges of the membrane cross-section. Top layer: blank space, middle: membrane, bottom layer: glass support.

**Figure 4:**
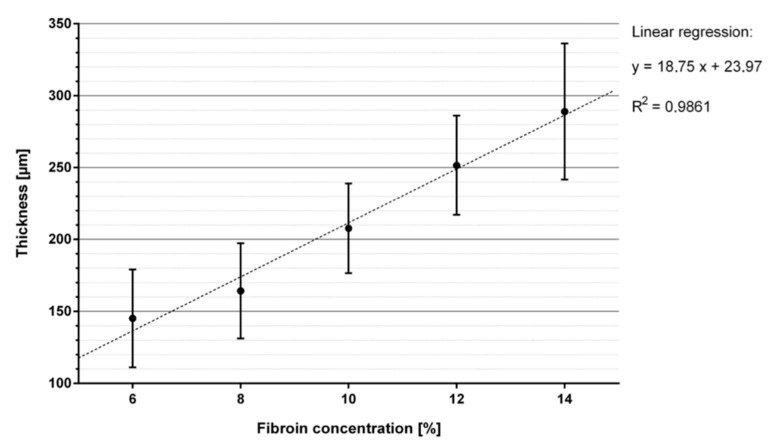
Mean value and standard deviation of membrane thickness with linear regression as a function of silk fibroin concentration.

Membranes exhibited different thicknesses within the cross-section. Table 2 presents the summarized results of the thickness analysis, based on measurements of seven membranes per casting concentration and four defined measuring points per membrane. Figure 3 presents the result of the thickness measurement at one spot of a SF12 membrane. A total of seven membranes were measured at four standardized spots each for their individual thickness and respective fibroin concentration level. Overall, membrane thickness increased nearly linearly with increasing fibroin concentration from approximately 145 μm at 6% fibroin (SF6) to approximately 289 μm at 14% fibroin (SF14) (Figure 4).

### 2.4. Mechanical Characterization

**Figure 5:**
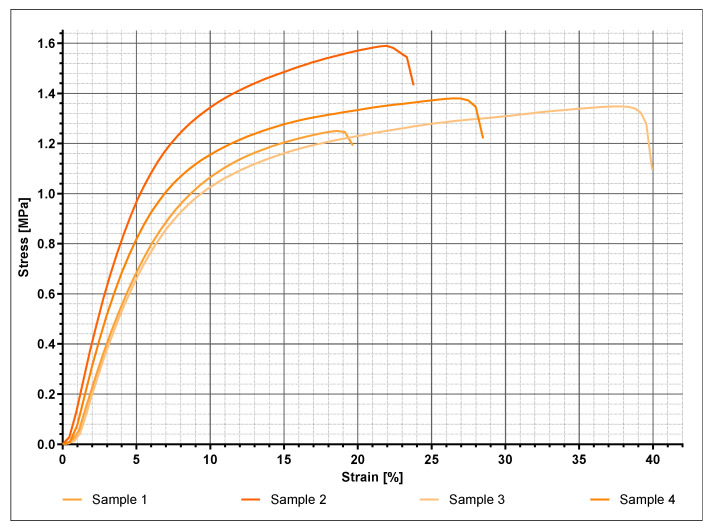
Stress–strain curves of four different SF10 samples. The varying starting points of the curves are related to measurements without preload, since travel in relation to clamping may partly be measured without force.

**Figure 6:**
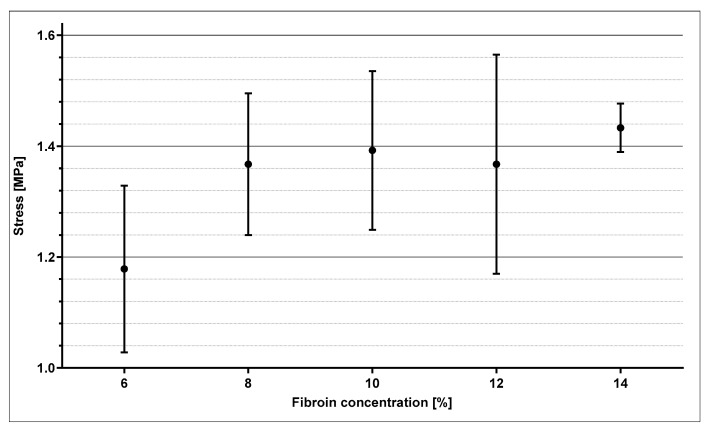
Mean value and standard deviation of maximum stress of the different membranes in relation to fibroin concentration.

**Figure 7:**
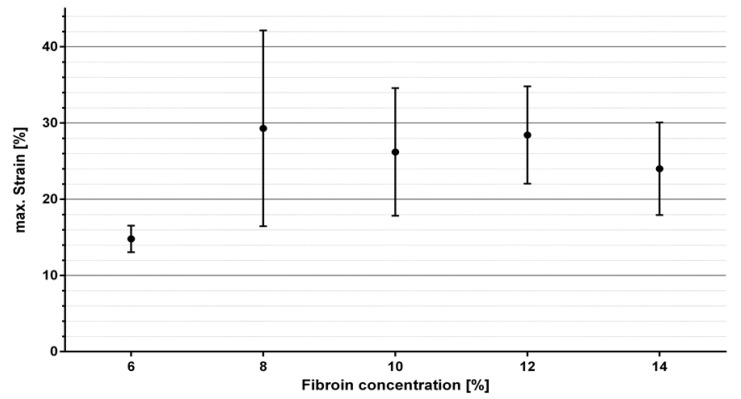
Mean value and standard deviation of maximum strain of the different membranes in relation to fibroin concentration.

The Membranes initially experienced a constant thinning out from both sides under increasing load, with them finally rupturing in exactly these areas. Figure 5 exemplarily indicates the stress–strain relationship of four SF10 membranes. The membranes displayed comparable elastic behavior, with noticeable differences in maximum tensile strength and associated strain, respectively. Within the first half towards maximum tensile strength, the linear relationship suggests elastic deformation. The further course of the mechanical response indicates a subsequent increasing proportion of plastic deformation. For the purpose of comparative evaluation concerning the various fibroin concentrations, the mean values of maximum stress and corresponding strain were statistically analyzed (Figure 6 and Figure 7).

The values displayed in Figure 6 and Figure 7 are derived from four samples used per fibroin concentration. Figure 6 demonstrates mean values and standard deviations of the maximum tensile strengths as a function of fibroin concentration. Except for SF6, nearly all measurements fluctuated among a maximum tensile strength of 1.4 MPa and formed a type of plateau. Only a slight tendency proposing increased maximum tensile stress with rising fibroin concentration could be surmised. Due to high standard deviation, no clear correlation could be demonstrated. The elongation values in Figure 7 displayed as a function of fibroin concentration exhibited a similar behavior. In this case, elongation could not be assigned towards any regularity or tendency and oscillated around values ranging from 14.8% to 30%.

### 2.5. Analysis of Transparency

**Figure 8:**
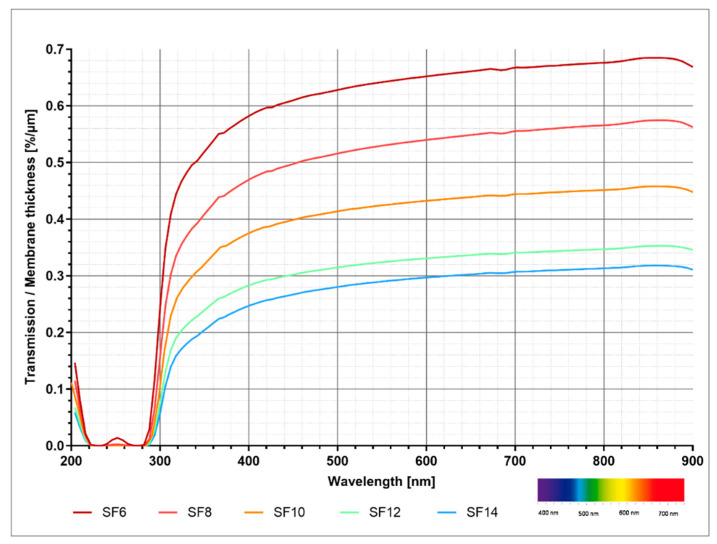
Adjusted degree of transmission in relation to the wavelength.

All membranes exhibited fairly small extinction rates between 0 and 0.2 in the visible and infrared range. In the UV range, increasing extinction showed two maxima (first between 230 nm and 240 nm with extinction of 3.5 to 3.8 and second between 265 nm and 275 nm with extinction of 3.45 to 3.95). A higher fibroin concentration was accompanied by higher extinction rates at all wavelengths. Figure 8 demonstrates the relationship between transparency and fibroin concentration, without the influence of membrane thickness, whereas interpretation of the data is strongly advised to be considered primarily qualitatively. Overall, membranes of lower fibroin concentration were accompanied by higher degrees of transmission and are therefore more permeable to electromagnetic radiation.

### 2.6. In Vitro Indirect Cytotoxicity

**Figure 9:**
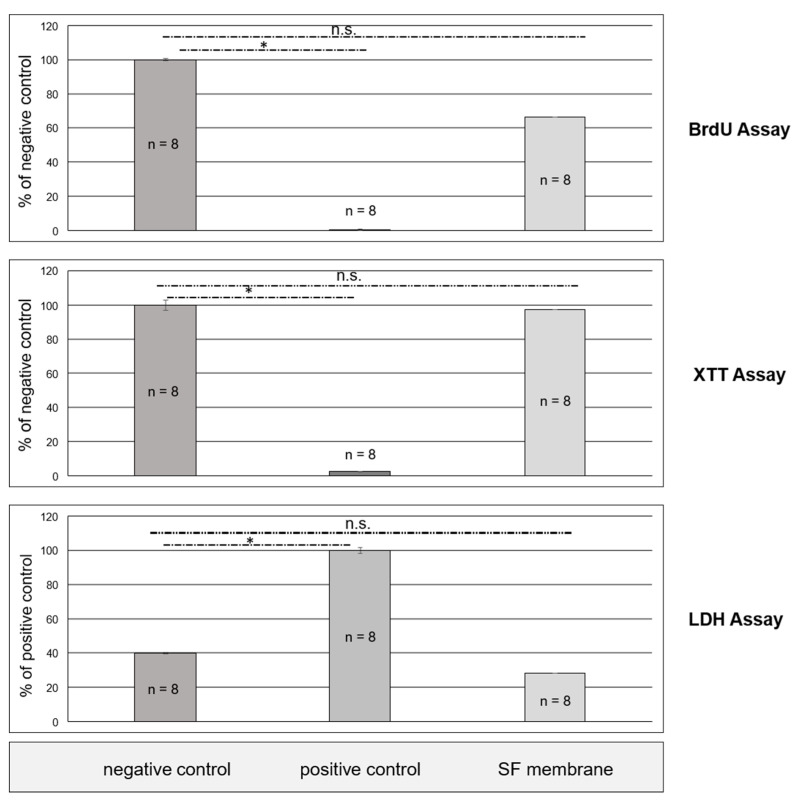
Results of indirect cytotoxicity tests on SF membrane with FA and CaCl_2_, following the ISO 10993-5, indicating statistical significance (*p* ≤ 0.05 (*); non significance (n.s.)).

Indirect in vitro assays were performed on SF10 to check whether the extractive compounds of fibroin membranes tinged with FA and CaCl_2_ could be considered as non-toxic and therefore suitable. Student’s *t*-test for independent samples was used for all tests in order to detect significant differences between the assessed groups. A *p* value of *p* < 0.05 was chosen as the confidence interval threshold where differences were considered to be significant. With reference to the XTT and BrdU tests, the fibroin membranes were compared in a pairwise manner either to the toxic positive control or the non-toxic negative control, respectively. In terms of the LDH assay, the procedure was reversed, with the positive control being used as the presentative basis. Essentially, all cell culture tests provided statistically significant evidence for absent cytotoxicity (Figure 9). Fibroin membranes stained with formic acid and CaCl_2_ were consistently situated within the range of the non-toxic negative control and markedly remote from the toxic positive control at all times. By implementing all three cell culture assays, a thorough assessment of fibroin membranes produced by implementing formic acid and CaCl_2_ in the manufacturing process could be performed. Fibroin derived from a novel production process could thus be introduced as a promising biomaterial. Neither suppression of cell metabolism, nor direct cell destruction could be detected in cells exposed to extracts of fibroin membranes at any given timepoint.

### 2.7. In Vitro Direct Cytotoxicity Testing

**Figure 10:**
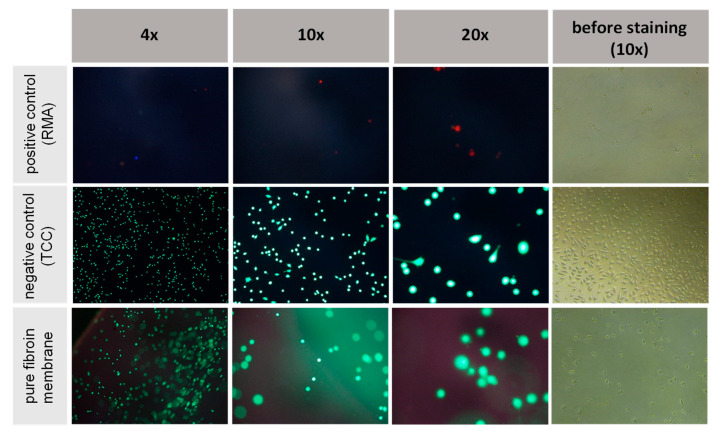
Live–dead staining of cells cultured on the surface of formic acid-based fibroin membranes.

To confirm the results of the indirect tests, the viability and adhesion of fibroblast cells was examined via direct cultivation on the silk membranes along with their respective controls and subsequent staining of the cultivated cells. Three samples (*n* = 3) of fibroin membranes fabricated via the formic acid and calcium chloride method and both reference materials were examined by fluorescent reflective light microscopy. Representative images were then selected for presentation, as seen in Figure 10. Following live–dead staining, all fibroblasts cultivated on both the fibroin membranes and the negative control (TCC) remained thoroughly vital, as clearly represented by green fluorescent and well-spread, spindle-shaped cells. Beside some single dead (red stained) cells, the toxic positive control (RMA) demonstrated almost no cells adhering to the surfaces at all, while the absence of red stained cells on the manufactured membranes also confirmed the supposed absence of toxicity. In summary, both the indirect and direct cytotoxicity tests indicated a good bio-response of the fibroin membranes tinted with formic acid and CaCl_2_, without occurrence of any cytotoxic effects.

## 3. Discussion

In the present study, an alternative approach employing formic acid and calcium chloride as solvents for the production of B. mori derived silk fibroin membranes was used. As previously reported, silk may be efficiently and simply dissolved with a process containing formic acid and CaCl_2_ solution [38,39,40]. Compared to other dissolution technologies, degummed silk transferred to FA/CaCl_2_ was readily soluble at room temperature. The regeneration process described in various other publications involves three steps, including the dissolution in an environment subjected to a high concentration of salts, followed by dialysis with distilled water and subsequent redissolving in alcohol or acidic solution [41,42]. Traditional solvents such as aqueous LiBr or CaCl_2_/ethanol/distilled water require time-consuming steps within the dissolution process and severely degrade the silk fibroin nanofibers [41,43]. Transparent and stable regenerated silk fibroin solution may also be obtained by dissolving the regenerated fibers in FA, as described previously by Um et al. [44]. The dissolution of silk fibroin using FA/CaCl_2_ has been thoroughly investigated in several publications, such as Liu. et al. and Zhang et al. [33,37]. While all these works have a similar sequence of process steps, they focus on different aspects of the FA/CaC_2_ manufacturing chain [33,34,35,36,37]. While papers such as Xue et al. and Yang et al. compared dissolved silk fibroin from various origins on a molecular level, Yang et al. and Liu et al. mainly analyzed FA/CaCl_2_-derived membranes under close examination of the different protein structures [35,36,37]. However, Zhang et al. previously reported on the influence of dissolution of silk fibroin in FA/CaCl_2_ on the molecular weight ratio and concluded that in comparison to common dissolution methods facilitating LiBr, CaCl_2_+EtOH, Ca(NO_3_)_2_+MeHO or LiSCN, among others, the FA/CaCl_2_ solvent rather showed decreased alterations in molecular weight [45].

In our study, we mainly focus on an applied research approach, evaluating both, a cost-effective method to manufacture silk fibroin membranes facilitating FA/CaCl_2_ as solvent and simultaneously assessing the most elemental requirements for medical devices, such as biocompatibility, by conducting in vitro assays based on the DIN EN ISO 10993 standard. Thus, we were able to establish an applicable and fast-forward protocol to manufacture silk fibroin devices based on dissolution of silk in FA/CaCl_2_. The suggested variation of processing sequences for silk fibroin membranes may hold several advantages in comparison to conventional methods, the most pronounced being cost effectiveness and reduced time consumption with regard to the translation towards an industrial manufacturing process. The ability to disintegrate into nanofibrils by applying solvents is another significant advantage of fibroin, thus enabling the formation of hierarchical fibril structures during further processing. Fibroin membranes produced in this study were characterized with regard to their surface, appearance and mechanical properties, as well as their respective transparency.

Mechanical properties are an essential characteristic of biomaterials and are fundamental with regard to their reactions and interactions with vital tissue. Results of mechanical tensile testing verified, that an increase in fibroin concentration is accompanied by an increase in tensile strength. Although no definitive statement can be made in terms of significance due to the incremental standard deviations, an increase in fibroin concentration does seem to generate ameliorated mechanical properties (tensile strength) of fibroin membranes. The mechanical properties of the membranes manufactured in this study lie slightly below those described in other studies [38,39,40]. However, the different manufacturing processes and test procedures applied in this study must be considered. For instance, there exists no published literature on membranes produced in an analogous manner, which were then assessed in a wetted state. Even if direct comparisons remain difficult, the comprehensive results suggest, that the membranes produced inhabit lower elasticity values than other wetted membranes, while their tension strength prevails at comparable magnitude [38,39]. This could be due to the manufacturing process used, as it may potentially lead to a formation of merely impaired beta sheet structures.

In addition to mechanical properties, optical properties are also of crucial importance for the function of fibroin membranes in biomedical engineering, especially when applied in corneal replacement. Depending on the intended use, here, transparency is either of primary or at least secondary importance. If envisioned as a type of cornea replacement, it is undoubtedly crucial that a non-functionalized membrane is permeable to visible light [41]. However, if used in other applications, as for example in wound dressings or as a separating measure in order to distinguish different tissue layers in surgery, transparency is of secondary importance. If used as a substitute for corneal tissue, a transmittance of 85% in the visible spectrum ranging from 400 nm to 780 m is required, whereas UV radiation, which is known to be harmful to the eye (approximately 100 nm to 400 nm), is highly undesirable [42]. The membranes produced in this study exhibited highly favorable optical properties, without undergoing further functionalization. Nevertheless, an increased concentration of fibroin led to a decrease in transmittance per thickness. Starting at wavelengths of approximately 520 nm, membranes of all fibroin concentrations expressed a transparency larger than 85%. SF6 membranes even illustrated transparency at 410 nm. Compared to other fibroin membranes described in the literature, the membranes manufactured in this study endorsed either comparable or improved optical properties in the range of visible light [40,41].

The superior physical properties possessed by silk as a biomaterial build upon complex hierarchical structures, which themselves are formed by nanoscale building blocks, namely beta-sheet nanocrystals and nanofibrils [44]. Many of the previously published regeneration processes result in the dissolution of these structures, which in particular have been recently recognized as the key to the high performance of native silk [46,47]. However, the dissolution of silk in FA/CaCl_2_ solution may also be regarded as advantageous, because the resulting fibers exhibit fibrillary structures of high hierarchical order, higher strength and increased extensibility, especially when compared to conventional manufacturing methods of native silk [48].

To ensure thorough biocompatibility, the physiological pH value (approximately pH 7.4) of surrounding tissues must not be influenced, as cell viability may consequently be impaired [49,50]. The process of membrane manufacturing pursued in this study requires comprehensive washing and neutralization. In our case, the entire process took two days and is thus an integral part of the actual production of the fibroin membranes. Final pH values were measured between 5.69 and 6.13, and therefore did not reach the physiological range. A possible cause may be the influence of the surrounding medium (1% AB solution with pH value of 6.04). An appropriate adaptation of the washing and neutralization processes is mandatory for further tests under more physiological conditions and should therefore be of primary interest in terms of additional investigation. Furthermore, supplemental experiments must be carried out in order to improve the reproducibility of the manufactured membranes, particularly with regard to uniform thickness and fewer impurities. This may be addressed by optimizing the manufacturing process in a way to ensure the balance of the resulting pH value and maintaining it within the physiological range. Once this has been achieved, membranes may be optimized with regard to various specific indications. If applied as a corneal alternate, the optical properties would have to be modified towards a higher transmittance for wavelengths in the visible light range and a lower transmittance for wavelengths within the UV range.

To further assess biocompatibility, additional in vitro experiments were carried out. For fibroin membranes generated via formic acid and CaCl_2_, cytotoxic effects were not detected in any of the test series. Especially LDH, a useful marker in cytotoxicity assessment of materials, was not detectable after incubation with fibroin membrane samples [51,52]. Also, live–dead staining showed no adverse side-effects of silk fibroin when in prolonged contact with fibroblasts. In this study, silk fibroin membranes manufactured using formic acid and CaCl_2_ did not have any negative impact on cell viability, cell proliferation and metabolic activity.

In summary, our test data addressing mechanical and optical properties as well as biocompatibility suggests membranes facilitating 10% casting concentration (SF10) as best case. Since we consider ophthalmological indications, among others, as potential translative applications, we were aiming on one hand on ensuring a high degree of transmission, while on the other hand optimizing mechanical properties, e.g., to prevent membrane damage during insertion or manipulation. In the present study, the SF10 variant represented the sweet spot of the before mentioned characteristics: above 10% fibroin casting concentration, the mechanical properties changed only marginally, but the transmission became noticeably worse.

## 4. Materials and Methods

### 4.1. Manufacture of Membranes

Raw silk was first degummed with 0.02 M Na_2_CO_3_ solution at 100 °C for 60 min and then washed thoroughly with deionized water for 10 min. Degummed silk fibroin was subsequently dried for 24 h and passed on for dissolution. The solvent was composed of 98% formic acid (VWR Chemicals, VWR International GmbH, Radnor, PA, USA) supplemented with 4% CaCl_2_ (calcium chloride, VWR Chemicals, VWR International GmbH, Radnor, PA, USA), which showed preferential (full) dissolution of fibroin in previous experiments [53].

The formic acid was added to different amounts of degummed fibroin in order to achieve different concentration levels of fibroin for the respective membranes (the term SF6/8/10/12/14 denotes fibroin concentration (SF6 = 6%; SF8 = 8%, SF10 = 10%, SF12 = 12% and SF14 = 14%, see Table 3). The resulting concentration levels of 6 to 14% were chosen in accordance with the literature, with these values being commonly used lower and upper thresholds for processing of fibroin solution and proper castability due to insufficient (<6%) or immoderate viscosity (>14%) [53]. To prepare the membranes, 5 mL of fibroin solution was casted into a petri dish (outer diameter: 100 mm) and left for 18 h to dry underneath a laminar flow bench. After 6 h of hydration using 50 mL of deionized water each, the membranes were carefully detached and forwarded through a complex washing and neutralization process (Figure 1). pH values were measured via corresponding pH electrode (pHenomenal 221, VWR Collection, VWR International GmbH, Leuven, Belgium) and pH meter (pH 1100 L, VWR Collection, VWR International GmbH, Leuven, Belgium) at defined timepoints within the washing and neutralization process. The number stated in the pH labels (pH 1–9) indicates the exact timepoint of pH measurement, which may be tracked according to the flow chart in Figure 1.

### 4.2. Characterization of Membranes

#### 4.2.1. Characterization of Surface and Structure

The structure of the membranes was analyzed via scanning electron microscopy (SEM) (Philips XL30 CP, Philips GmbH, Hamburg, Germany). After undergoing drying for 18 h, two separated pieces were fixed to a holder either in horizontal or upright orientation and subsequently sputtered (Au) in argon medium for 90 s at a vacuum of 3 × 10^−1^ bar (sputter coater S150B, Edwards, London, UK). Three images were taken for each fibroin concentration, respectively: an overview image with a 30× magnification, a close-up image of the membrane surface with a 2000× magnification and a close-up cross-sectional image. Images were saved and then analyzed with ImageAccess Standard program (IMAGIC BILDVERARBEITUNG AG, Glattbrugg, Switzerland).

#### 4.2.2. Determination of Membrane Thickness

Membrane thickness was analyzed via light microscopy (LEICA DM 2500 M, Leica Camera AG, Wetzlar, Germany) with a 100× magnification. Until subjected to the respective measurements, membranes were maintained in a wetted state placed in deionized water supplemented with antibiotics 1% Antibiotic (Pen Strep (Gibco Life Technologies, Carlsbad, CA, USA) with 10.00 units/mL Penicillin and 10.00 μg/mL Streptomycin). Membranes were positioned on a vertically oriented microscope slide in order to enable the examination of the membrane cross-section. Respective images were taken at four spots previously marked on the slide, which themselves were equally distributed among the diagonal of the membrane. Images were saved and then analyzed with ImageAccess Standard program (IMAGIC BILDVERARBEITUNG AG, Glattbrugg, Switzerland).

#### 4.2.3. Mechanical Characterization

Four membranes per fibroin concentration were analyzed, whereas samples that had ruptured before testing or those that had slipped out of the holding clamps were excluded from further evaluation. Mechanical testing was performed using a zwickiLine materials testing machine Z2.5 (Zwick GmbH & Co. KG, Ulm, Germany). Strips measuring 20 mm in width were cut from the middle area of the wetted membranes, consecutively dabbed off and then fixed in a Vulkollan-coated type 8033 sample holder (Zwick GmbH & Co. KG, Ulm, Germany) with a maximum force of Fmax 200 N. Samples were then pulled at 10 mm/min at a holding length of 50 mm without a respective preload. Force (N) was measured as a function of elongation.

#### 4.2.4. Analysis of Transparency

Five samples per fibroin concentration were examined. Transparency was analyzed by photo spectroscopy (Spectrophotometer V-550, Rev. 1.00, JASCO Labor-und Datentechnik GmbH, Gross-Umstadt, Germany). Rectangular strips measuring 25 mm × 8 mm were cut from the middle area of the wetted membranes and subsequently transferred into a quartz glass Suprasil^®^ QS cuvette 200–2500 nm (Hellma GmbH & Co. KG, Müllheim, Germany). Extinction (*E*) was measured against a reference cuvette in visible and UV spectral range at wavelengths between 200 nm and 900 nm. Transmittance was then calculated. The transmission degree (*T*) describes the quotient of the outgoing (*I*) and incoming light intensity (*I*_0_).

$$T=\frac{I}{I_{0}}$$

In spectroscopy measurements, the extinction at a specific wavelength is determined as the negative decadic logarithm of the transmission which can be calculated using following formula.

$$E=lg\left(\frac{I_{0}}{I}\right)=-\mathrm{lg}\left(T\right)$$

$$T=10^{-E}$$

#### 4.2.5. In Vitro Indirect Cytotoxicity Testing

Novel biomaterials must pass certain toxicity tests based on part 5 of the standard ISO 10993 (ISO 10993 Biological Evaluation of Medical Devices. Standard. International Organization for Standardization (ISO), Geneva, Switzerland) in order to ensure biocompatibility and safety. Thus, corresponding indirect in vitro assays were performed to check for compatibility of extractive compounds gained from fibroin membranes tinged with formic acid and CaCl_2_. Ninety-six-well plates were seeded with 1 × 104 L929 cells per well in 100 µL cell culture medium and then incubated for 24 h under cell culture conditions. The cell culture medium was subsequently discarded and 100 µL of membrane extract was applied to each well. The cells were incubated for another 24 h and consecutively exposed to the BrdU and XTT tests, whereas the respective supernatants were subjected to lactate dehydrogenase (LDH) testing. Similar assays were performed for all extracts, except for the control prepared in order to detect any possible assay interferences, which was formulated without cells. Blank controls (medium alone without cells) were subtracted from the absorbance values in order to adjust the readout at all times.

##### Bromodeoxyuridine/5-Bromo-20-Deoxyuridine (BrdU)-Assay

The BrdU (colorimetric) test kid (Roche Diagnostics GmbH, Mannheim, Germany) was executed as described in the manufacturer’s instructions. Briefly, cells were labeled with BrdU for 2 h under cell culture conditions and subsequently fixed for 30 min at room temperature with FixDenat reagent.

Then, the fixed cells were incubated for 1 h with anti-BrdU-peroxidase (POD) antibody and washed 3 times for 5 min in washing buffer. Immune complexes were detected after subsequent substrate reaction with tetramethyl-benzidine (TMB) (20 min at room temperature) followed by the addition of 25 μL 1 M H_2_SO_4_ for the purpose of reaction termination via scanning multi-well spectrophotometer (ELISA reader) equipped with filters for 450 nm and 690 nm (reference wavelength).

##### Sodium 3,30-[1(Phenylamino)Carbonyl]-3,4-Tetrazolium]-3is(4-Methoxy-6-Nitro) Benzene Sulfonic acid Hydrate (XTT)-Assay

The Cell Proliferation Kit II for XTT-assay (Roche Diagnostics GmbH, Mannheim, Germany) was executed as described in the manufacturer´s instructions. Briefly, the electron-coupling reagent was mixed with XTT labeling reagent (1:50 dilution) and 50 μL of the corresponding mixture was then added to the cells. After 4 h of incubation under cell culture conditions, substrate conversion was quantified by measuring the absorbance of 100 μL aliquots in a fresh 96-well plate via scanning multi-well spectrophotometer (ELISA reader) equipped with filters for 450 nm and 650 nm (reference wavelength).

##### Lactate Dehydrogenase (LDH) Assay

The LDH-Cytotoxicity Assay Kit II (BioVision, Milpitas, CA, USA) was executed as described in the manufacturer´s instructions. Briefly, 10 μL of the respective cell supernatants were incubated with 100 μL LDH reaction mix for 30 min at room temperature. After the addition of stopping solution, absorbance was measured via scanning multi-well spectrophotometer (ELISA reader) equipped with filters for 450 nm and 650 nm (reference wavelength). Paired t-tests were performed to analyze differences between each group for all cytotoxicity tests.

#### 4.2.6. In Vitro Direct Cytotoxicity Testing

For direct cell culture testing, 12 well plates were seeded with 2.4 × 105 cells in 1 mL cell culture medium. Cells were seeded directly onto the surface of the test specimens and incubated under cell culture conditions for 24 h before performing direct assay procedures. To perform live–dead cell staining on the surfaces of the specimens, 60 µL propidium iodide (PI) stock solution, consisting of Phosphate-Buffered Saline (PBS) supplemented with 50 µg/mL PI were added to each well (12 well plate). In addition, 500 µL fresh fluorescein diacetate (FDA) working solution (20 µg/mL in PBS from 5 mg/mL FDA in acetone stock solution) were also added for each well. The wells were subsequently incubated for 3 min at room temperature (RT), followed by rinsing the specimens in preheated PBS and immediately examining them with a fluorescence microscope equipped with a filter for parallel detection of red and green fluorescence.

## 5. Conclusions

In this study, we introduced a simple and inexpensive method for the production of silk fibroin membranes by using FA/CaCl_2_ solution as an initial solvent followed by a cascaded rinsing series in water and buffer solutions. To avoid the conventional, expensive dialysis process, an alternative washing and neutralization process was investigated. Manufactured membranes displayed favorable physical and mechanical properties, but require improvement concerning pH optimization and prolonged production time. The membranes produced in this study exhibit a compact structure, displaying a proportional correlation of most properties along with increasing fibroin concentration, as, e.g., membrane thickness and transparency. Accordingly, the performance of a fibroin membrane may be predictably controlled by means of fibroin concentration variation. Based on our findings, a concentration level of 10% (SF10) showed the best combination of characteristics, as a further increase of fibroin concentration could not improve the mechanical properties of the membranes, but was detrimental in terms of transmission, which is of utmost importance when for example aiming for ophthalmologic applications. This facilitated and novel process for the manufacturing of fibroin membranes may not only be advantageous in terms of simplicity, cost-effectiveness and efficiency in comparison to conventional methods, but it may also be categorized as less disruptive due to the conservation of the nanofibril structures. Fibroin membranes prepared via formic acid and calcium chloride dissolution displayed thorough biocompatibility in all cell culture tests performed within this study. Nevertheless, further research must be pursued in order to establish silk fibroin as a recognized engineering biomaterial not only in medicine, but to ensure its suitability for a broad variety of other applications.

## Figures and Tables

**Table 1:** pH values during washing and neutralization. pH 1 to pH 9, indicating the exact time of measurement, which can be tracked using the flow chart in Figure 1. AB = 1% Antibiotic (Pen Strep (Gibco Life Technologies, Carlsbad, CA, USA) with 10.00 units/mL Penicillin and 10.00 μg/mL Streptomycin) pH of 1% in deionized water: 6.04. SF digit encodes the fibroin concentration (SF6 = 6%; SF8 = 8%, SF10 = 10%, SF12 = 12%, and SF14 = 14%).

Membrane	pH 1	pH 4 after Washing Process	pH 5 after 16.5 h in 1% AB Water	pH 7 after Neutralization	pH 9 after 17 h in 1% AB Water
SF6	6.67	6.81	5.32	7.30	6.13
SF8	6.51	6.75	5.16	7.02	5.69
SF10	5.92	6.73	5.04	7.08	6.08
SF12	4.46	6.70	4.95	7.52	6.00
SF14	4.50	6.72	4.89	7.40	6.12

**Table 2:** Results of membrane thickness measurements of seven membranes per casting concentration and four measuring points per membrane.

Sample	Thickness Measurement Mean Value (µm)	Standard Deviation (± µm)
SF6	145.12	32.8294
SF8	164.209	31.5686
SF10	207.63	30.1487
SF12	251.558	33.7116
SF14	288.974	45.3632

**Table 3:** Chemical composition of the facilitated fibroin solutions.

Name	Fibroin Concentration [%] (*w*/*v*)	CaCl_2_ Concentration in Formic Acid [%] (*w*/*v*)
SF6	6	4
SF8	8	4
SF10	10	4
SF12	12	4
SF14	14	4

## Abbreviations

FA	Formic acid
CaCl_2_	Calcium chloride
SEM	Scanning electron microscopy
UV	Ultraviolet
